# Mental Health Outcomes Among *Travestis* and Transgender Women in Brazil: A Literature Review and a Call to Action for Public Health Policies

**DOI:** 10.3390/ijerph22070977

**Published:** 2025-06-20

**Authors:** David R. A. Coelho, Ana Luiza N. Ferreira, Willians Fernando Vieira, Alex S. Keuroghlian, Sari L. Reisner

**Affiliations:** 1 Division of Public and Community Psychiatry, Massachusetts General Hospital, Boston, MA 02114, USA; 2 Harvard T.H. Chan School of Public Health, Boston, MA 02115, USA; 3 Instituto Fernandes Figueira, Fundação Oswaldo Cruz, Rio de Janeiro 22250-020, RJ, Brazil; 4 Instituto e Centro de Pesquisas São Leopoldo Mandic, Faculdade São Leopoldo Mandic, Campinas 13045-755, SP, Brazil; 5 Departamento de Anatomia, Instituto de Ciências Biomédicas, Universidade de São Paulo, São Paulo 05367-000, SP, Brazil; 6 The Fenway Institute, Fenway Health, Boston, MA 02215, USA; 7 Department of Psychiatry, Harvard Medical School, Boston, MA 02215, USA; 8 Department of Psychiatry, Massachusetts General Hospital, Boston, MA 02114, USA; 9 Department of Epidemiology, Harvard T.H. Chan School of Public Health, Boston, MA 02215, USA; 10 Department of Epidemiology, University of Michigan, Ann Arbor, MI 48109, USA

**Keywords:** *travesti*, transgender women, mental health, public policies, Brazil

## Abstract

*Travestis* and transgender women in Brazil face a disproportionate burden of mental health conditions, exacerbated by structural discrimination, violence, and social exclusion. This narrative review synthesizes evidence on the prevalence of depression, anxiety, suicidality, and substance use among *travestis* and transgender women in Brazil, and examines intersecting social and health disparities. We searched PubMed, Embase, and PsycINFO in April 2025, identifying peer-reviewed studies in English or Portuguese reporting mental health outcomes or associated social determinants of health in this population. Thirty-one studies across twelve different cities (*n* = 7683) were included and grouped into two thematic domains. Reported prevalence ranged from 16–70.1% for depression, 24.8–26.5% for anxiety, and 25–47.3% for suicidality. Substance use was also highly prevalent, with studies reporting high rates of alcohol (21.5–72.7%), tobacco (56.6–61.6%), cannabis (19–68.9%), and cocaine/crack (6–59.8%) use. Discrimination, violence, economic hardship, and HIV were consistently associated with psychological distress and barriers to care. These findings underscore the urgent need to integrate mental health, gender-affirming care, and HIV services into Brazil’s Unified Health System (*Sistema Único de Saúde–SUS*), strengthen anti-discrimination and violence-prevention policies, and adopt inclusive public health strategies that prioritize the leadership and lived experiences of transgender, nonbinary, and gender diverse people, particularly amid rising political threats to gender-affirming care.

## 1. Introduction

Transgender, nonbinary, and gender diverse people across the globe experience a disproportionate burden of mental health conditions due to pervasive societal stigma, discrimination, and structural inequities [1,2,3]. In Brazil, an estimated 2% of the adult population—nearly three million people—identify as transgender, nonbinary, and gender diverse people [4]. These communities face health disparities that are further compounded by widespread social exclusion and violence [5]. Notably, Brazil has accounted for approximately one-third of all reported global murders of transgender, nonbinary, and gender diverse people over the past 17 years [6,7]. In parallel, HIV prevalence among *travestis*—a culturally specific gender identity within the transfeminine spectrum in Latin America [8]—and transgender women in Brazil remains alarmingly high, ranging from 24% to 54%, underscoring the urgent need for inclusive, equity-oriented public health strategies [9,10,11,12].

Evidence consistently shows that transgender, nonbinary, and gender diverse people are at elevated risk for depression, anxiety, and suicidality compared to cisgender populations [13,14,15]. A meta-analysis reported pooled prevalence rates of 50% for lifetime suicidal ideation and 29% for lifetime suicide attempts among transgender, nonbinary, and gender diverse people [16]. In another study involving over 600 transgender, nonbinary, and gender diverse adults, 72.2% had received a lifetime diagnosis of depression, and 73% had received a lifetime diagnosis of anxiety [17]. These disparities are often explained through the minority stress model, which posits that chronic exposure to structural and interpersonal discrimination contributes to sustained psychological distress [18,19]. Studies across multiple settings have further demonstrated associations between societal stigma and increased risk of poor mental health outcomes and barriers to healthcare access [20,21,22].

In Brazil, these mental health risks are exacerbated by systemic barriers within the public healthcare system [23]. The Brazilian Unified Health System (*Sistema Único de Saúde*–SUS), founded on principles of universality, integrality, and equity, offers free and universal healthcare [24]. However, the implementation of gender-affirming care and mental health services for transgender, nonbinary, and gender diverse people remains limited and unevenly distributed across regions [25]. Although gender-affirming care, including hormone therapies and gender-affirming surgeries, is formally recognized within SUS, access is largely concentrated in a small number of specialized urban centers with long waitlists [26]. In addition, mental health services tailored to this marginalized population are rarely integrated into primary care or HIV prevention and treatment programs, despite the syndemic overlap between HIV, psychological distress, and structural vulnerability [5]. Inadequate provider training, discrimination in healthcare settings, and logistical barriers further restrict access to care and reinforce existing inequities [5].

Despite increasing global recognition of the health disparities affecting transgender, nonbinary, and gender diverse communities [27], there remains a lack of consolidated evidence in Brazil that synthesizes mental health outcomes and their intersecting social determinants. Many existing reviews examined mental health across broader LGBTQIA+ communities but they have not focused specifically on *travestis* and transgender women [5,26,28,29,30], or they examined only HIV-related outcomes [31,32]. To address this gap, we conducted a narrative review to: (1) provide an overview of the prevalence of depression, anxiety, suicidality, and substance use among *travestis* and transgender women in Brazil, focusing on these communities given the predominance of existing studies; (2) examine intersecting social and health disparities, including discrimination, violence, economic exclusion, and HIV and other health outcomes; and (3) propose public health policy strategies grounded in equity, integration, and expanded access to mental health and gender-affirming care within SUS for this highly marginalized population.

## 2. Materials and Methods

### 2.1. Search Strategy

We conducted a narrative review of articles reporting on mental health outcomes among *travestis* and transgender women in Brazil. The search was carried out across three databases—PubMed, Embase, and PsycINFO—without date restrictions. We developed search terms to capture a broad range of mental health outcomes and used combinations of terms related to gender identity (e.g., “*travesti*,” “transgender,” “transgender woman,” “transgender women,” “trans woman,” “trans women,” “gender diverse”) and mental health (e.g., “depression,” “anxiety,” “suicidality,” “suicidal,” “suicide,” “substance,” “substance-related disorder,” “substance use disorder,” “alcohol,” “tobacco,” “cannabis,” “marijuana,” “cocaine,” “crack,” “amphetamine,” “methamphetamine,” “opioid,” “heroin,” “inhalant,” “ecstasy,” “polysubstance” “mental disorder,” “mental health”), along with “Brazil.” The search was first conducted in December 2023 and updated in April 2025.

### 2.2. Eligibility Criteria

We included studies that met the following criteria: (1) included *travestis* or transgender women; (2) examined mental health outcomes, including depression, anxiety, suicidality, and/or substance use; or reported on social determinants of health, including discrimination, physical, psychological, and sexual violence, economic hardship, and HIV and other health outcomes; (3) presented prevalence estimates for mental health outcomes and effect measures for social determinants of health; (4) were peer-reviewed; and (5) were published in English or Portuguese. We excluded case reports, case series, qualitative-only studies, abstracts, protocols, and studies where outcomes were reported alongside transgender men and nonbinary people and could not be extracted separately for *travestis* and transgender women.

### 2.3. Study Selection and Data Extraction

All records were screened by title and abstract, followed by full-text review of potentially eligible articles. Discrepancies were resolved through discussion and consensus. A standardized data extraction form was used to collect the following information: author(s), year of publication, study location(s), sample size, participant characteristics, mental health outcomes and/or social determinants of health assessed, and key prevalence or association estimates (e.g., odds ratios [OR], risk ratios [RR], prevalence rates [PR]).

### 2.4. Data Synthesis

Given the heterogeneity in study design, measurement tools, and outcome definitions, a narrative synthesis approach was adopted. Results were grouped into two main domains: (1) mental health outcomes, including depression, anxiety, suicidality, and substance use, and (2) intersecting social and health disparities, including discrimination, physical, psychological, and sexual violence, economic hardship, and HIV and other health outcomes. When different publications reported distinct outcomes from the same study cohort, we included each publication separately and noted the overlapping cohorts. Outcomes were reported as described in the original studies, which varied in timeframe (e.g., lifetime vs. recent) and in operationalization. In some cases, studies reported the presence of any symptom or substance use, while others used standardized scales with defined cutoffs or severity categories. This variability was preserved to reflect the methodological diversity across the included literature. For ease of reference, studies were organized alphabetically by first author’s name in both summary tables. Key findings were summarized in tabular format, and effect estimates were reported as ranges where applicable. No quantitative meta-analysis was conducted due to the narrative nature of the review and substantial heterogeneity across studies.

## 3. Results

### 3.1. Overview of Included Studies

**Figure 1:**
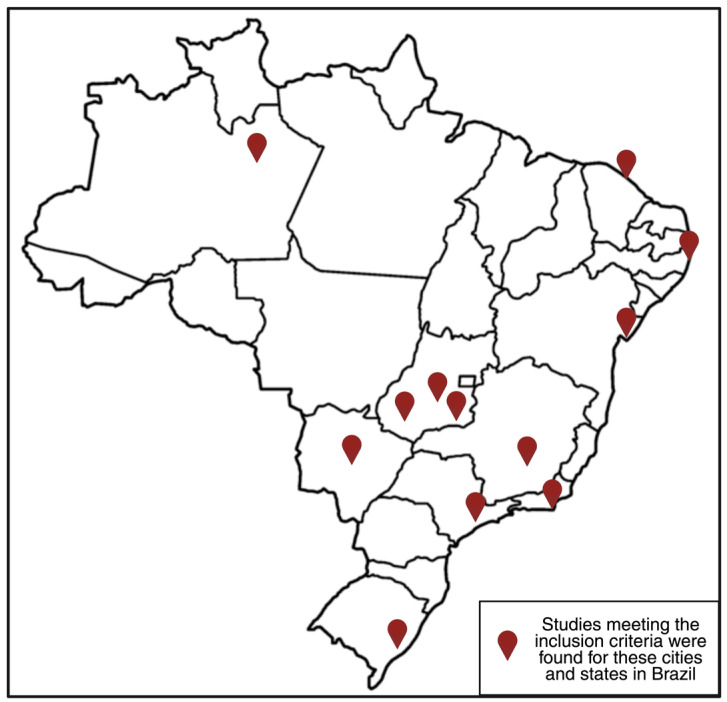
Geographic distribution of studies on mental health and social determinants among *travestis* and transgender women across Brazilian cities and states.

We included a total of thirty-one studies (k = 31) [9,10,11,12,33,34,35,36,37,38,39,40,41,42,43,44,45,46,47,48,49,50,51,52,53,54,55,56,57,58,59]. Ten studies (k = 10) examined mental health outcomes among *travestis* and transgender women in Brazil [12,33,34,35,36,37,38,39,40,41]. Specifically, six (k = 6) focused on depression [12,33,34,35,36,37], two (k = 2) on anxiety [35,36], four (k = 4) on suicidality [35,36,38,39], and six (k = 6) on substance use [12,35,37,39,40,41]. Twenty-six studies (k = 26) reported on intersecting social and health disparities [9,10,11,12,33,35,38,39,42,43,44,45,46,47,48,49,50,51,52,53,54,55,56,57,58,59], including five (k = 5) on discrimination [42,43,44,45,46], nine (k = 9) on violence [33,35,38,39,42,44,47,48,49], two (k = 2) on economic difficulties [12,43], and fourteen (k = 14) on HIV and health disparities [9,10,11,12,50,51,52,53,54,55,56,57,58,59]. After removing studies that analyzed overlapping cohorts, the total number of unique participants across studies was 7683 *travestis* and transgender women. The studies were conducted across multiple Brazilian cities: São Paulo (SP) (k = 15) [11,35,37,39,41,43,44,45,47,49,50,51,53,55,56], Salvador (BA) (k = 12) [10,11,33,34,41,43,44,46,47,51,52,55], Rio de Janeiro (RJ) (k = 8) [12,38,42,48,54,57,58,59], Campo Grande (MS) (k = 6) [9,11,41,43,47,55], Porto Alegre (RS) (k = 6) [11,36,41,43,47,55], Manaus (AM) (k = 5) [11,41,43,47,55], Belo Horizonte (MG) (k = 3) [34,44,51], Fortaleza (CE) (k = 3) [10,33,46], Recife (PE) (k = 3) [10,33,46], Goiânia (GO) (k = 1) [40], Itumbiara (GO) (k = 1) [40], and Jataí (GO) (k = 1) [40]. Figure 1 illustrates the geographic distribution of included studies across cities and states in Brazil.

### 3.2. Mental Health Outcomes Among Travestis and Transgender Women in Brazil

Substantial mental health challenges have been documented among *travestis* and transgender women in Brazil, particularly regarding depression, anxiety, suicidality, and substance use [12,33,34,35,36,37,38,39,40,41]. Table 1 shows these studies on mental health outcomes.

The burden of depression is especially pronounced among Brazilian *travestis* and transgender women, with studies reporting rates between 16% and 70.1% [12,33,34,35,36,37]. For example, in a multi-city study conducted in Fortaleza, Recife, and Salvador, 70.1% of participants exhibited symptoms of major depressive disorder (MDD) [33]. Similarly, a study across Belo Horizonte, Salvador, and São Paulo found that 69.6% reported depressive symptoms, with 44.6% experiencing severe cases [34]. Other studies found depressive symptoms in 57.8% of participants in Rio de Janeiro [12], 19.1% and 16% in São Paulo [35,37], and 16.6% in Porto Alegre [36]. While prevalence rates vary by region and methodology, they consistently indicate rates well above those observed in the general Brazilian population, where the estimated prevalence of depression is approximately 10.2% [60].

Anxiety and suicidality are also critical public health concerns among *travestis* and transgender women in Brazil. Anxiety symptoms were reported in 24.8% [36] to 26.5% [35] of participants in studies from São Paulo and Porto Alegre, nearly triple the national prevalence of 9.3% [61]. Suicidality was also particularly alarming, ranging from 25% to 47.25% for suicidal ideation and 27.25% to 39.8% for suicide attempts [35,36,38,39]. For instance, in Rio de Janeiro, 47.25% of participants had experienced suicidal ideation, and 27.25% had attempted suicide [38]. In Porto Alegre, 46.4% had a history of suicidal ideation and 31.2% of suicide attempt [36]. In São Paulo, suicidal ideation was reported by 25% of participants, with 31.2% reporting prior suicide attempts [35], while another study found a 39.8% prevalence of suicide attempts [39].

Substance use is also highly prevalent among Brazilian *travestis* and transgender women, with reported rates ranging from 1.3% for less common substances like hallucinogens or hypnotics to 72.7% for alcohol [12,35,37,39,40,41]. Notably, only tobacco and alcohol are legal substances in Brazil, and other commonly reported substances, such as cannabis, cocaine, and crack, are illegal for recreational use [62]. However, recent legal changes have decriminalized the possession of small amounts of cannabis for personal use under specific limits, while cannabis-based medications remain permitted in certain medical contexts [62]. Across studies, alcohol use ranged from 21.5% to 72.7% [12,35,37,39,40,41], tobacco from 56.6% to 61.6% [12,40,41], cannabis from 19% to 68.9% [12,35,37,40,41], and cocaine/crack from 6% to 59.8% [12,37,40,41]. For comparison, national estimates in the general population indicate a smoking prevalence of 11.3% [63], heavy episodic drinking at 17.1% [64], cannabis use at 2.1% [65], and crack-cocaine use at 2.2% [66]. Lifetime use of multiple substances, including inhalants, stimulants, and other drugs, was also frequent, with some studies reporting overall illicit or recreational drug use in 46% to 66.4% of participants [35,37,39,41]. Specifically, in a study conducted across Goiânia, Itumbiara, and Jataí, high rates of at-risk substance use were observed: marijuana (68.9%), tobacco (59.8%), cocaine/crack (59.8%), and binge drinking (56.6%) [40]. In São Paulo, alcohol use in the past year was reported by 72.7% of participants, while cannabis (46.8%) and stimulant use (44.4%) were also prevalent [35]. Another São Paulo-based study reported 30% alcohol use and 46% illicit or recreational drug use, including marijuana (19%), cocaine (15%), and crack (6%) [37]. In Rio de Janeiro, problematic use of tobacco (56.6%), cannabis (28.9%), cocaine (23.8%), and alcohol (21.5%) was also documented [12]. In addition, a multi-city study spanning Campo Grande, Manaus, Porto Alegre, Salvador, and São Paulo found high lifetime use of alcohol (65.5%), tobacco (61.6%), marijuana (52%), cocaine (42.6%), inhalants (14.6%), crack (13.9%), amphetamines/ecstasy (11.8%), and other substances, including hypnotics, hallucinogens, and opioids (1.3–13.1%) [41].

Taken together, these studies illustrate a mental health crisis among *travestis* and transgender women in Brazil. The consistently elevated prevalence rates across multiple indicators—depression, anxiety, suicidality, and substance use—signal a pattern of psychological distress shaped by social exclusion and systemic inequities [12,33,34,35,36,37,38,39,40,41]. Compared to population-level estimates for mental health disorders in Brazil [60,61], these rates are substantially higher, reinforcing the need for focused, culturally responsive mental health interventions for this marginalized community.

### 3.3. Intersecting Social and Health Disparities Faced by Travestis and Transgender Women in Brazil 

In Brazil, the mental health of *travestis* and transgender women is shaped by a constellation of intersecting social determinants that exacerbate psychological distress and limit access to care. These include discrimination, violence, economic marginalization, and HIV-related disparities [9,10,11,12,33,35,38,39,42,43,44,45,46,47,48,49,50,51,52,53,54,55,56,57,58,59]. Table 2 shows these studies on intersecting social and health disparities.

Discrimination remains a widespread and compounding structural barrier. In Rio de Janeiro, 96% of participants reported experiencing discrimination, which was significantly associated with increased depressive symptoms [42]. In São Paulo, a study using the Intersectional Discrimination Index found that each unit increase in anticipated discrimination was associated with higher odds of severe psychological distress (adjusted odds ratio [aOR] = 2.13, 95% confidence interval [CI]: 1.57–2.89) and suicidality (aOR = 1.44, 95% CI: 1.08–1.93) [45]. In a multi-site study across Fortaleza, Recife, and Salvador, discrimination was associated with lower odds of seeking medical care (OR = 0.29; 95% CI: 0.14–0.63) and HIV testing (OR = 0.41; 95% CI: 0.22–0.78) [46]. Among adolescent *travestis* and transgender women (15–19 years) in Belo Horizonte, Salvador, and São Paulo, 50% reported experiencing discrimination six times or more [44]. Similarly, in the national *TransOdara* study, 85% of participants across Campo Grande, Manaus, Porto Alegre, Salvador, and São Paulo reported some form of discrimination [43]. Alarmingly, in this study, 23% had been arrested, yet fewer than 1% were placed in dedicated LGBTQIA+ detention spaces, highlighting institutional neglect and structural violence [43].

Experiences of violence further compound these disparities. In Rio de Janeiro, 52% of *travestis* and transgender women reported physical violence, and 42% reported sexual violence—both of which were significantly associated with increased depressive symptoms [42]. Another study from the same city found that physical violence was associated with increased suicidal ideation (adjusted prevalence ratio [aPR] = 1.37; 95% CI: 1.09–1.71) and suicide attempts (aPR = 1.92; 95% CI: 1.28–2.88) [38]. Childhood abuse was also identified as a critical factor in Rio de Janeiro: emotional abuse—defined as experiences such as being insulted or cursed at by parents, or hearing them express regret that the child had been born—was associated with suicide attempts (OR = 9.00; 95% CI: 1.13–71.34) [48]. In the same study, emotional neglect—characterized by not feeling supported or loved, or being special—was associated with self-injurious behavior (OR = 11.64; 95% CI: 2.35–57.5) [48]. In the *TransOdara* study, which included participants from five Brazilian cities, 14.2% of *travestis* and transgender women reported having experienced physical violence [47], and 51% reported being forced to have sex [43]. Similarly, high rates were observed in São Paulo, where 45.1% of participants had suffered sexual violence [39]. Another study from the same city reported that 62% had experienced physical violence and 45% sexual violence [49]. In addition, lifetime sexual violence was associated with a 56% reduction in HIV viral suppression (adjusted risk ratio [aRR]: 0.44, 95% CI: 0.24–0.79) in that study [49]. In another analysis from São Paulo, sexual violence was associated with both suicidal ideation and suicide attempts (OR = 1.69; 95% CI: 1.18–2.44) [35], and among adolescent (15–19 years) *travestis* and transgender women in Belo Horizonte, Salvador, and São Paulo, 45% reported having suffered sexual violence [51]. Lastly, in northeastern cities including Fortaleza, Recife, and Salvador, both physical (OR = 2.09; 95% CI: 1.20–3.67) and sexual violence (OR = 2.06; 95% CI: 1.15–3.68) were associated with mild to moderate symptoms of MDD [33].

Economic hardship is another central factor. In the *TransOdara* study, 37.6% of *travestis* and transgender women reported unstable housing, only 8.4% were formally employed, and 57% earned up to one minimum wage [43]. Additionally, 73.7% had engaged in transactional sex, and 21.3% identified sex work as their main income source [43]. In a Rio de Janeiro study, 78.6% of participants reported current or past engagement in sex work, and 62% earning less than USD 10 per day [12].

HIV-related disparities further deepen health inequities. HIV prevalence was reported at 54% in Rio de Janeiro [12], 34% in the *TransOdara* study across five cities [11], 24.5% in a multicenter study conducted across Fortaleza, Recife, and Salvador [10], and 24% in Campo Grande [9]. Two studies in Salvador found HIV prevalence ranging from 9% to 24.3%, with gender-based discrimination associated with testing positive for HIV (OR = 8.65; 95% CI: 1.45–51.59) [46]. In São Paulo, HIV prevalence was disproportionately high among younger *travestis* and transgender women (ages 18–24) (RR = 3.85; 95% CI: 1.24–12.93) compared to older peers [53]. Two studies in Rio de Janeiro and São Paulo found that a history of sex work was associated with newly diagnosed HIV cases (OR = 30.7; 95% CI: 3.5–267.3 and RR = 5.90; 95% CI: 1.71–26.62, respectively) [53,54]. Among participants living with HIV in the *TransOdara* study, 42% had a detectable viral load, despite most being on antiretroviral therapy (ART) [55]. Younger age (PR = 2.26; 95% CI: 1.13–4.51), poor housing (PR = 2.72; 95% CI: 1.30–5.68), and self-rated poor/very poor mental health (PR = 1.70; 95% CI: 1.08–2.66) were associated with unsuppressed viremia [55]. Moreover, discrimination emerged as a key impediment to consistent engagement in HIV care services. A mediation analysis in São Paulo found that anticipated stigma was associated with reluctance to report new symptoms to healthcare providers among *travestis* and transgender women living with HIV (aOR = 7.42; 95% CI: 1.93–28.5) [56]. Lastly, in terms of health disparities in São Paulo, 36.8% reported current use of nonprescribed hormones due to factors such as financial hardship, with the highest rates among those aged 18–25 (49.1%) [50].

Among those not living with HIV, *travestis* and transgender women in Rio de Janeiro aged 18–24 had lower knowledge of HIV pre-exposure prophylaxis (PrEP) (OR = 0.5; 95% CI: 0.3–0.8) and HIV post-exposure prophylaxis (PEP) (OR = 0.5; 95% CI: 0.3–0.9), and were more likely to engage in high-risk behaviors, including at-risk substance use (OR = 1.8; 95% CI: 1.1–2.9) and unprotected sex (OR = 1.8; 95% CI: 1.1–3.0) [58]. In a multicenter study across Belo Horizonte, Salvador, and São Paulo, 79.3% of *travestis* and transgender women aged 15–19 reported condomless anal sex in the past six months [51]. In Rio de Janeiro, a study found higher odds of early loss to follow-up in PrEP care among *travestis* and transgender women (aOR = 2.8; 95% CI: 1.6–4.8) [59]. In another study from Rio de Janeiro, although 85.4% of participants were retained in care over 48 weeks, only 48.6% had high PrEP adherence based on tenofovir levels in dried blood spots [57]. Specifically, participants aged 18–24 had more missing study visits (aOR: 8.76; 95% CI: 2.09–36.7) compared to those aged 35+, and stimulant use was also associated with missed visits (aOR: 4.99; 95% CI: 1.37–18.1) [57].

Together, these findings demonstrate how structural and interpersonal factors, including discrimination, violence, economic hardship, HIV vulnerability, and health disparities, interact to exacerbate mental health conditions and restrict access to care among *travestis* and transgender women in Brazil [9,10,11,12,33,35,38,39,42,43,44,45,46,47,48,49,50,51,52,53,54,55,56,57,58,59]. Compared to the general population, this group faces disproportionate exposure to psychosocial stressors and structural barriers [9,10,11,12,33,35,38,39,42,43,44,45,46,47,48,49,50,51,52,53,54,55,56,57,58,59], underscoring the need for integrated, affirming, and equity-oriented policy interventions.

## 4. Discussion

**Figure 2:**
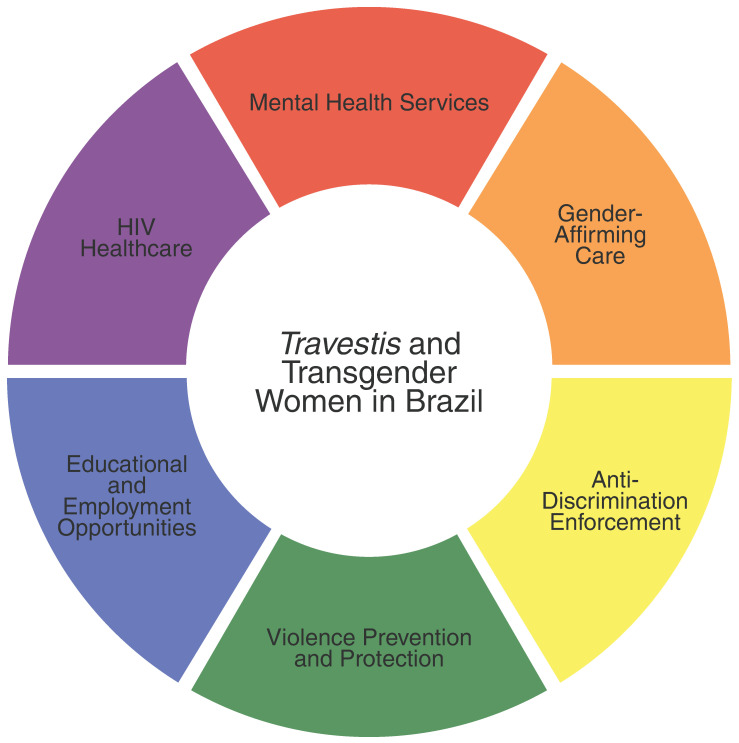
Key policy areas to improve health equity for *travestis* and transgender women in Brazil.

This review synthesized evidence concerning mental health outcomes and intersecting social determinants affecting *travestis* and transgender women in Brazil. Across multiple studies and cities, elevated rates of depression, anxiety, suicidality, and substance use were consistently reported [12,33,34,35,36,37,38,39,40,41], alongside structural disparities including discrimination, violence, economic exclusion, and HIV vulnerability [9,10,11,12,33,35,38,39,42,43,44,45,46,47,48,49,50,51,52,53,54,55,56,57,58,59]. These findings reveal a syndemic environment driven by systemic inequities and underscore the urgent need for integrated mental health and gender-affirming care strategies. Table 3 outlines key policy recommendations based on this review and their corresponding stakeholders, and Figure 2 provides a visual representation of the main policy areas.

A key implication is the need to expand mental health services, including psychotherapy, within Brazil’s public healthcare system. *Travestis* and transgender women face multiple barriers to care, including stigma, lack of trained providers, and limited access to specialized services [67]. Although full integration within SUS remains a logistical challenge, co-locating mental health support in existing gender-affirming care settings, HIV clinics, and primary care settings offers a feasible and equity-oriented approach. Initiatives such as those implemented by *Fundação Oswaldo Cruz (Fiocruz)*, which offer integrated, community-based services tailored to transgender, nonbinary, and gender diverse people, demonstrate the feasibility and impact of such models in Brazil [12]. Moreover, enhancing referral systems, particularly in underserved regions, and investing in provider training are essential for improving access and retention [68,69].

In parallel, expanding access to gender-affirming care is critical. Although SUS formally recognizes services such as hormone therapies and gender-affirming surgeries, access remains limited and unevenly distributed, with services concentrated in large urban centers and often requiring complex referrals and long wait times [25,26]. These access gaps are exacerbated by recent political efforts to restrict transgender, nonbinary, and gender diverse rights nationally and globally, limiting healthcare protections and threatening progress toward equity and inclusion [70]. Robust evidence shows that gender-affirming care is associated with significant reductions in depression, anxiety, and suicidality, as well as improvements in quality of life and psychological well-being [71,72]. Ensuring that gender-affirming services are not only protected but expanded within SUS is essential to promoting mental health equity and upholding the human rights of *travestis* and transgender women in Brazil. This is particularly urgent in the context of a global political climate marked by growing hostility toward transgender, nonbinary, and gender diverse communities [70,73]. For example, in countries like the United States, recent legislative rollbacks have restricted access to gender-affirming care [70]. Similarly, Brazil has experienced periods of political regression, including increased anti-gender rhetoric, attempts to dismantle LGBTQIA+ protections, and, more recently, efforts to prohibit the use of pubertal suppression and hormone therapies for transgender, nonbinary, and gender diverse young people [73,74]. Therefore, it is essential to anchor public health strategies in a human rights framework to ensure access to care and to resist the politicization of health.

Structural discrimination must also be directly addressed. While anti-discrimination laws exist in Brazil, enforcement remains inconsistent across healthcare, education, and employment [75]. Strengthening legal protections and establishing accountability mechanisms are critical to preventing mistreatment and denial of care. National data show that *travestis* and transgender women may be more likely to access Basic Health Units (UBS), yet experiences of discrimination, especially from reception staff, significantly reduce continued service use [76]. This highlights the need for respectful, inclusive clinical environments and ongoing provider training. In addition, public awareness campaigns and digital tools like the *Dandarah* app, used by over 4000 LGBTQIA+ people to report violence and access psychosocial support [77], illustrate how technology can be leveraged to improve safety and strengthen connections to care, particularly in a context of pervasive violence against transgender, nonbinary, and gender diverse people in Brazil [6,7].

Equally important is addressing socioeconomic exclusion. As detailed in this review, many *travestis* and transgender women face chronic financial insecurity and rely on sex work as their primary—and often sole—source of income due to limited access to formal employment [12,55]. Advancing education and workforce inclusion is essential to breaking this cycle. Initiatives, such as *PreparaNem*, a university preparatory program for LGBTQIA+ students, create pathways to higher education and long-term mobility [78]. More recently, affirmative action policies at several Brazilian universities have emerged, following sustained advocacy from organizations such as the *Associação Nacional de Travestis e Transexuais* (ANTRA) and other LGBTQIA+ civil society groups [73,79]. These efforts should be complemented by inclusive policies at earlier educational levels, including primary and secondary schools, to reduce dropout rates and ensure safer, more supportive learning environments for transgender, nonbinary, and gender diverse young people. Employment programs like *TransEmpregos*, which connect transgender, nonbinary, and gender diverse people to inclusive job opportunities, also show promise in reducing poverty and improving mental health outcomes [80]. These programs deserve broader support and scale-up through partnerships with government and private employers.

In addition, strengthening HIV prevention and care must remain a national public health priority [81], particularly given the disproportionate burden of HIV among transgender, nonbinary, and gender diverse populations [82]. In addition, depression, substance use, and stigma can all compromise adherence to ART, exacerbating morbidity and inequities [46,52,56,57,58]. Integrating mental health support into HIV care settings and implementing routine mental health screening during HIV testing, prevention, and treatment could significantly improve outcomes. Expanding access to and awareness of PrEP, PEP, and community-based HIV testing is especially urgent among young transgender, nonbinary, and gender diverse people and those engaged in sex work, who face substantial barriers to care [54,57,58]. For example, a recent scoping review further identified low perceived risk, fear of confidentiality breaches, and younger age as key barriers to HIV rapid testing among *travestis* and transgender women in Brazil, while autonomy and favorable testing environments were cited as facilitators, especially in the context of self-testing [31]. These findings reinforce the value of youth-specific, privacy-preserving, and community-led strategies to improve HIV testing uptake and retention in care.

Ultimately, these policies must place transgender, nonbinary, and gender diverse populations at their core, centering their lived experiences and leadership. Community-based participatory research and inclusive governance, with ANTRA and other LGBTQIA+ groups engaged throughout, enhance relevance and methodological rigor. Funding frameworks must prioritize sustained support for community-driven advocacy and capacity building, while institutionalizing mechanisms for transgender, nonbinary, and gender diverse representation in decision-making bodies. As depicted in Figure 2, this people-centered approach not only upholds human rights and agency but is indispensable for advancing health equity and building an inclusive, resilient public health system.

This review has several strengths. To our knowledge, it is the first national synthesis focused specifically on mental health outcomes among *travestis* and transgender women in Brazil, integrating mental health data with structural vulnerabilities, such as discrimination, violence, economic exclusion, HIV, and other health disparities. The inclusion of studies from different regions allows for a more comprehensive snapshot of the health needs and lived experiences of this marginalized population. Additionally, the review brings together both epidemiological data and public policy implications, providing an evidence-based foundation for focused mental health and gender-affirming care strategies in the Brazilian context.

However, some limitations should be noted. The included studies varied in methodology, sample size, and outcome measures, limiting comparability. Most relied on cross-sectional data, with few longitudinal analyses. Moreover, many samples were drawn from urban settings and may not fully reflect the experiences of rural or Indigenous transgender, nonbinary, and gender diverse populations. This review was also conducted as a narrative review rather than a systematic or scoping review and focused exclusively on *travestis* and transgender women, given the predominance of research on this group in Brazil, thereby excluding studies on transgender men and nonbinary people. In addition, qualitative studies were not included, though they offer valuable insight into lived experiences. Future research should prioritize longitudinal and intervention studies to better understand causal pathways between structural violence and mental health outcomes, and to evaluate the effectiveness of integrated service models and policy reforms. Expanding gender identity data collection in national health surveys and improving surveillance systems will also be critical to increasing the visibility of Brazilian *travestis* and transgender women in health policy and research.

## 5. Conclusions

The mental health landscape for *travestis* and transgender women in Brazil presents significant challenges. While the studies included do not encompass all regions, they provide valuable insights and highlight the urgent need for tailored public health policies. It is a call to action for creating an inclusive and equitable environment while ensuring that the health and dignity of *travestis* and transgender women are not only recognized but are actively championed as a measure of Brazil’s commitment to equality and human rights.

## Figures and Tables

**Table 1:** Mental health outcomes among *travestis* and transgender women in Brazil.

Author (Year)	City (s)	Sample Size	Main Findings
Almeida et al. (2022) [33]	Fortaleza	864	Symptoms of major depressive disorder (70.1%)
	Recife		
	Salvador		
Bassichetto et al. (2023) [39]	São Paulo	113	Alcohol use any (72.5%)
			Illicit substance use (66.4%)
			Suicide attempt (39.8%)
Ferreira et al. (2019) [12]	Rio de Janeiro	322	Depressive symptoms (57.8%)
			Problematic use of tobacco (56.6%)
			Problematic use of cannabis (28.9%)
			Problematic use of cocaine (23.8%)
			Problematic use of alcohol (21.5%)
Magalhães et al. (2024) [40]	Goiânia	440	Marijuana (68.9%)
	Itumbiara		Cocaine/crack (59.8%)
	Jataí		Tobacco (59.8%)
			Binge drinking (56.6%)
Medeiros et al. (2023) [34]	Belo Horizonte	56	Overall depressive symptoms (69.6%)
	Salvador		Severe depressive symptoms (44.6%)
	São Paulo		Mild/moderate depressive symptoms (25%)
Mota et al. (2024) [41]	Campo Grande	1317	Alcohol (65.5%)
	Manaus		Tobacco (61.6%)
			Marijuana (52%)
	Porto Alegre		Cocaine (42.6%)
			Inhalants (14.6%)
	Salvador		Crack (13.9%)
			Amphetamines/ecstasy (11.8%)
	São Paulo		Other substances, including hypnotics, hallucinogens, and opioids (1.3–13.1%)
Rafael et al. (2021) [38]	Rio de Janeiro	345	Suicidal ideation (47.25%)
			Suicide attempt (27.25%)
Reis et al. (2021) [35]	São Paulo	763	Alcohol use in the past year (72.7%)
			Cannabis use in the past year (46.8%)
			Stimulant drug use in the past year (44.4%)
			Suicide attempt (31.2%)
			Anxiety (26.5%)
			Suicidal ideation (25%)
			Depression (19.1%)
Sabino et al. (2021) [37]	São Paulo	106	Any illicit/recreational drugs use (46%)
			Alcohol use (30%)
			Marijuana (19%)
			Cocaine (15%)
			Depression (16%)
			Crack (6%)
Silva et al. (2021) [36]	Porto Alegre	111	Suicidal ideation (46.4%)
			Suicide attempt (31.2%)
			Anxiety symptoms (24.8%)
			Depressive symptoms (16.6%)

**Table 2:** Intersecting social and health disparities faced by *travestis* and transgender women in Brazil.

Author (Year)	City (s)	Sample Size	Factor (s)	Main Findings
Amarante et al. (2023) [56]	São Paulo	113	HIV and health disparities	Fear of public mistreatment was associated with increased difficulty reporting new symptoms to healthcare providers (aOR = 7.42; 95% CI: 1.93–28.5)
Almeida et al. (2022) [33]	Fortaleza	864	Physical and sexual violence	Sexual (OR = 2.06; 95% CI: 1.15–3.68) and physical violence (OR = 2.09; 95% CI: 1.20–3.67) were associated with increased mild/moderate symptoms of major depressive disorder
	Recife			
Barros et al. (2024) [55]	Campo Grande	425	HIV and health disparities	42% had detectable viral load; associated with poor mental health (PR = 1.70; 95% CI: 1.08–2.66), poor housing (PR = 2.72; 95% CI: 1.30–5.68), and younger age (PR = 2.26; 95%CI: 1.13–4.51)
	Manaus			
	Porto Alegre			
	Salvador			
	São Paulo			
Bassichetto et al. (2023) [39]	São Paulo	113	Sexual violence	45.1% had suffered sexual violence
Cesar et al. (2024) [9]	Campo Grande	152	HIV and health disparities	24% were living with HIV
Costa et al. (2021) [50]	São Paulo	790	Health disparities	36.8% used nonprescribed hormones; use was higher among young *travestis* and transgender women 18–25 (49.1%)
Depret et al. (2025) [48]	Rio de Janeiro	139	Psychological violence	Emotional abuse in childhood was associated with suicide attempts (OR = 9.00; 95% CI: 1.13–71.34), and emotional neglect in childhood was associated with self-injury behaviors (OR = 11.64; 95% CI: 2.35–57.5)
Dourado et al. (2024) [11]	Campo Grande	1317	HIV and health disparities	34% were living with HIV
	Manaus			
	Porto Alegre			
	Salvador			
	São Paulo			
Echeverría-Guevara et al. (2023) [59]	Rio de Janeiro	1463	HIV and health disparities	*Travestis* and transgender women had significantly higher odds of early loss to follow-up for PrEP (aOR 2.8; 95% CI: 1.6–4.8)
Ferreira et al. (2019) [12]	Rio de Janeiro	322	Economic difficulties	78.6% were current or past sex workers, and 62% earned below USD 10/day
			HIV and health disparities	54% were living with HIV
Grinsztejn et al. (2017) [54]	Rio de Janeiro	345	HIV and health disparities	Newly diagnosed cases of HIV were associated with a history of sex work (OR = 30.7; 95% CI: 3.5–267.3)
Koreitem et al. (2025) [45]	São Paulo	392	Discrimination	Higher anticipated discrimination was associated with psychological distress (aOR = 2.13; 95% CI: 1.57–2.89) and suicidality (aOR = 1.44; 95% CI: 1.08–1.93)
Jalil et al. (2022) [57]	Rio de Janeiro	130	HIV and health disparities	Only 48.6% had high PrEP adherence; missed visits were associated with younger age (18–24 years) (aOR = 8.76; 95% CI: 2.09–36.7) and stimulant use (aOR = 4.99; 95% CI: 1.37–18.1)
Leite et al. (2021) [46]	Fortaleza	864	Discrimination	Discrimination was associated with a reduced likelihood of medical visits (OR = 0.29; 95% CI: 0.14–0.63) and HIV testing (OR = 0.41; 95% CI: 0.22–0.78)
	Recife			
	Salvador			
Leite et al. (2022) [52]	Salvador	127 and 166 (Two studies)	HIV and health disparities	HIV prevalence ranged from 9% to 24.3%, respectively, and gender-based discrimination was associated with testing positive for HIV (OR = 8.65; 95% CI: 1.45–51.59)
Leite (2024) [10]	FortalezaRecifeSalvador	864	HIV and health disparities	24.5% were living with HIV
Luz et al. (2022) [42]	Rio de Janeiro	489	Discrimination	96% had suffered discrimination
			Physical violence	52% had suffered physical violence
			Sexual violence	42% had suffered sexual violence
Magno et al. (2024) [47]	Campo Grande	1317	Sexual violence	51% had suffered sexual violence
	Manaus			
	Porto Alegre		Physical violence	14.2% had suffered physical violence
	Salvador			
	São Paulo			
Rafael et al. (2021) [38]	Rio de Janeiro	345	Physical violence	Physical violence was associated with increased suicidal ideation (aPR = 1.37; 95% CI: 1.09–1.71) and suicide attempt (aPR = 1.92; 95% CI: 1.28–2.88)
Reis et al. (2021) [35]	São Paulo	763	Sexual violence	Sexual violence was associated with increased suicidal ideation and suicide attempt (OR = 1.69; 95% CI: 1.18–2.44)
Rosário et al. (2024) [51]	Belo Horizonte	121	HIV and health disparities	79.3% of *travestis* and transgender women aged 15–19 reported condomless anal sex in the past 6 months
	Salvador			
	São Paulo			
Ryngelblum et al. (2023) [44]	Belo Horizonte	72	Discrimination	50% had suffered discrimination six times or more
	Salvador		Sexual violence	45% had suffered sexual violence
	São Paulo			
Veras et al. (2021) [50]	São Paulo	545	HIV and health disparities	Higher prevalence of HIV among *travestis* and transgender women aged 18–24 (RR = 3.85; 95% CI: 1.24–12.93) and those who engaged in sex work in the last month (RR = 5.90; 95% CI: 1.71–26.62)
Veras et al. (2024) [55]	São Paulo	113	Physical and sexual violence	62% and 45% had suffered physical and sexual violence, respectively, and lifetime sexual violence was associated with a 56% reduction in viral suppression (aRR = 0.44; 95% CI: 0.24–0.79)
Veras et al. (2024) [43]	Campo Grande	1317	Discrimination	85% reported experiencing discrimination
	Manaus			
	Porto Alegre			
	Salvador		Economic difficulties	57% earned ≤ 1 minimum wage and 21.3% reported sex work as main income source
	São Paulo			
Wilson et al. (2021) [58]	Rio de Janeiro	345	HIV and health disparities	Younger ages (18–24 years) were associated with lower odds of PrEP awareness (OR = 0.5; 95% CI: 0.3–0.8) and PEP knowledge (OR = 0.5; 95% CI: 0.3–0.9), as well as higher odds of at-risk substance use (OR = 1.8; 95% CI: 1.1–2.9) and unprotected sex (OR = 1.8; 95% CI: 1.1–3.0)

aOR: adjusted odds ratio; CI: confidence interval; OR: odds ratio; aPR: adjusted prevalence ratio; aRR: adjusted risk ratio; RR: risk ratio; PR: prevalence ratio; PrEP: HIV pre-exposure prophylaxis; PEP: HIV post-exposure prophylaxis.

**Table 3:** Integrated policy recommendations and key stakeholders for comprehensive transgender mental healthcare in Brazil.

Policy Area	Recommendations	Key Stakeholders
Mental health services	Expand mental health services, including psychotherapy, within centers offering gender-affirming care, HIV prevention and care, and primary care settings. Strengthen referral pathways in SUS to improve access, especially in remote areas. Prioritize provider training and sensitization to enhance trust and care navigation for transgender, nonbinary, and gender diverse people.	SUS, Ministry of Health, healthcare professionals, professional medical associations, local governments, and advocacy groups.
Gender-affirming care	Integrate gender-affirming care into SUS primary care settings and promote continuous training for healthcare professionals in LGBTQIA+ care.	SUS, Ministry of Health, healthcare professionals, professional medical associations, medical education institutions, local governments, and advocacy groups.
Discrimination	Enforce anti-discrimination laws and policies to protect transgender, nonbinary, and gender diverse people in healthcare, education, and employment. Develop and promote public awareness campaigns to combat stigma and discrimination against transgender, nonbinary, and gender diverse people.	Ministry of Justice and Public Security, SUS, Ministry of Health, Ministry of Education, Ministry of Labour and Employment, local governments, and advocacy groups.
Violence	Promote the *Dandarah* app and other initiatives aimed at enhancing safety and providing avenues for reporting incidents. Strengthen law enforcement training on LGBTQIA+ issues to ensure proper handling of cases involving transgender, nonbinary, and gender diverse people.	Ministry of Justice and Public Security, SUS, Ministry of Health, technology developers, law enforcement, social media platforms, local governments, and advocacy groups.
Educational opportunities	Establish affirmative policies across all levels of education, from primary schools to universities, to support transgender, nonbinary, and gender diverse students. Expand initiatives such as *PreparaNem* and other preparatory programs. Integrate LGBTQIA+ awareness into school curriculums to reduce stigma and promote inclusion.	Ministry of Education, universities, schools, NGOs, local governments, and advocacy groups.
Employment opportunities	Develop job training programs and enhance NGO initiatives like *TransEmpregos* to foster economic empowerment. Implement anti-discrimination policies in workplaces.	Ministry of Labour and Employment, Employment agencies, private sector, labor unions, educational institutions, NGOs, local governments, and advocacy groups.
HIV healthcare	Expand the use of PrEP and PEP, particularly among younger *travestis* and transgender women and those engaged in sex work. Maintain high rates of ART adherence while providing mental health support for those living with HIV. Integrate HIV healthcare with mental health services and substance use screening with a harm reduction approach.	SUS, Ministry of Health, healthcare professionals, community health workers, local governments, and advocacy groups.

SUS: Sistema Único de Saúde (Unified Health System); NGO: Non-governmental organization; PrEP: HIV pre-exposure prophylaxis; PEP: HIV post-exposure prophylaxis; ART: antiretroviral therapy.

## Data Availability

No new data were created or analyzed in this study. Data sharing is not applicable to this article.
